# Co-distribution of Light At Night (LAN) and COVID-19 incidence in the United States

**DOI:** 10.1186/s12889-021-11500-6

**Published:** 2021-08-04

**Authors:** Yidan Meng, Vincent Zhu, Yong Zhu

**Affiliations:** Department of Environmental Health Sciences, Yale University School of Public Health, New Haven, CT 06520 USA

**Keywords:** Light at night (LAN), COVID-19

## Abstract

**Background:**

Light at night (LAN) as a circadian disruption factor may affect the human immune system and consequently increase an individual’s susceptibility to the severity of infectious diseases, such as COVID-19. COVID-19 infections spread differently in each state in the United States (US). The current analysis aimed to test whether there is an association between LAN and COVID-19 cases in 4 selected US states: Connecticut, New York, California, and Texas.

**Methods:**

We analyzed clustering patterns of COVID-19 cases in ArcMap and performed a multiple linear regression model using data of LAN and COVID-19 incidence with adjustment for confounding variables including population density, percent below poverty, and racial factors.

**Results:**

Hotspots of LAN and COVID-19 cases are located in large cities or metro-centers for all 4 states. LAN intensity is associated with cases/1 k for overall and lockdown durations in New York and Connecticut (*P* < 0.001), but not in Texas and California. The overall case rates are significantly associated with LAN in New York (*P* < 0.001) and Connecticut (*P* < 0.001).

**Conclusions:**

We observed a significant positive correlation between LAN intensity and COVID-19 cases-rate/1 k, suggesting that circadian disruption of ambient light may increase the COVID-19 infection rate possibly by affecting an individual’s immune functions. Furthermore, differences in the demographic structure and lockdown policies in different states play an important role in COVID-19 infections.

**Supplementary Information:**

The online version contains supplementary material available at 10.1186/s12889-021-11500-6.

## Background

Mammalian circadian rhythms, controlled by a neurological master clock located in the suprachias matic nucleus (SCN) and the peripheral clocks of somatic cells, regulate a number of biological and physiological processes including the human immune system [1, 2]. Because immune responses play a major role in fighting against virus infections [3], a disrupted circadian rhythm may adversely influence immune functions and consequently increase virus infectivity and its ability to replicate inside hosts [4–6].

Circadian disruptions can be caused by sleep deprivation, night shift work, frequent air traveling, circadian gene alterations, and light at night (LAN) exposures [7–10]. Sleep deprivation has been associated with increased susceptibility to gut infection [11]. In addition, a higher incidence and severity of respiratory infections has been reported among night shift workers [12]. These findings support a significant relationship between disrupted circadian rhythms and an individual’s increased vulnerability to infectious diseases and suggest that excess risk could also be observed among individuals with high LAN exposures for the infection of COVID-19 [2], a coronavirus causing the global pandemic in 2020.

There are also studies analyzing LAN exposure and various cancer types, such as breast cancer, prostate cancer, thyroid cancer, and non-Hodgkin Lymphoma [7, 9, 13, 14]. Both global and regional studies have shown that there is a significant association between intensity of light at night and breast cancer [7, 14–16]. These findings suggest that LAN as a circadian disruption can influence the immune system and hormone releases, and in turn affect an individual’s susceptibility to infectious diseases as well.

Light at night comes from either ambient light or indoor artificial light exposures. Excessive exposure to LAN may generate light pollution that causes adverse effects on immune functions [17] and alters circadian gene functions in the SCN [13]. City-level LAN intensity can be measured by using the U.S. Defense Meteorological Satellite Program (DMSP). In the present analysis, we investigate whether exposure to LAN is associated with COVID-19 incidence in major cities in four selected US states: Connecticut, New York, Texas, and California, each of which represent different geological locations.

## Methods

### Data sources

We obtained COVID-19 cases and testing data from local health departments. Specifically, we obtained COVID-19 data for Connecticut from the Connecticut State Department of Public Health (https://data.ca.gov/dataset/covid-19-cases), for New York State from the Open NY Program (https://data.ny.gov/browse?tags=covid-19), for Texas from Texas Health and Human Services (https://dshs.texas.gov/coronavirus/additionaldata.aspx), and for California from the California Open Data Portal (https://data.ca.gov/dataset/covid-19-cases). All of the above databases are open to the public and no permissions are required to access these data. In summary, we obtained 62 data points for New York State, 167 data points for Connecticut, 56 data points for California, and 254 data points for Texas, based on the data availability for either county or town level. Data of COVID-19 cases were categorized in 3 groups: duration of overall (March 20th, 2020 to August 4th, 2020), period of lockdown, and period of reopening in each state according to local state policy on the government websites [18–22].

LAN intensity data was extracted from satellite images of nighttime light intensity created by the NASA Earth Observatory [23]. We also collected demographic data, including factors of income and poverty, race/ethnicity, and population density. County level demographic data for California, Texas, and New York was obtained from the US Census (https://data.census.gov), and town level demographic data for Connecticut was obtained from local public health departments (https://portal.ct.gov/DPH). County and town level boundaries data was obtained from local transportation or planning departments (http://gis.ny.gov/gisdata; https://data.ct.gov; https://gis-txdot.opendata.arcgis.com; https://gisdata-caltrans.opendata.arcgis.com).

### Geographic information system (GIS) mapping

ArcMap (https://desktop.arcgis.com/en/arcmap) was used to generate visualized hotspot or density maps for COVID-19 case rate data and LAN data. The *Kernel Density* (KD) in ArcMap 10.8.1 was used to calculate density from neighborhood features to create a smooth raster layer from points or polylines. The search radius was calculated by spatial configuration and the total number of points in the dataset, and equal breaks were used for symbology for Connecticut, Texas, and California. Natural breaks were used for symbology for New York because the LAN level in New York City is much higher than other cities in New York and natural breaks can better present data with large differences of inherent groups. Because of the huge differences in LAN data of New York City compared to other cities in New York State, we specifically analyzed the spatial pattern of LAN and COVID-19 case rates and performed a Geographically Weighted Regression (GWR). The Spatial Autocorrelation (Global Moran’s I) tool was used to test spatial patterns (clustered, dispersed, or random) of points for cases/1 k during the lockdown, reopening, and overall periods for New York State. GWR was used to understand regional variation of geo-data.

### Statistically analysis

To analyze the correlation between LAN intensities and COVID-19 case rates per 1000 people, we built multiple linear regression models with variables of nonwhite rate, percent below poverty, and population density from the US Census, 2015: ACS 5-Years Estimates Subject Tables (https://data.census.gov). Population density is a very important factor that showed moderate or strong positive correlation with the number of COVID-19 infections [24–26]. Controlling the factor of population density in the regression models can help to eliminate the effect of human-to-human contact on COVID-19 infection. Multiple regression models were performed using SAS 9.4 and RStudio.

## Results

To represent the different regions of the U.S., we included four states, Connecticut, New York, California, and Texas in the current analysis. Generally, hotspots of LAN data were located in large cities or metro-centers for all four states tested, such as Hartford, CT, New York City, NY, Dallas, TX, and San Francisco, CA.

The maximum LAN intensity calculated by ArcMap tool was 254.68, 254.68, 235.14 and 190.30 in New York State, Connecticut, Texas, and California, respectively. The mean LAN intensity was 92.31 in Connecticut and around 40 in the other three states. The maximum COVID-19 case -rate was in Texas (around 67 cases/1 k people) among all four states, followed by California (around 47 cases/1 k people), New York (around 43 cases/1 k people), and lowest in Connecticut (around 27 cases/1 k people) at the time of data collection.

**Fig. 1:**
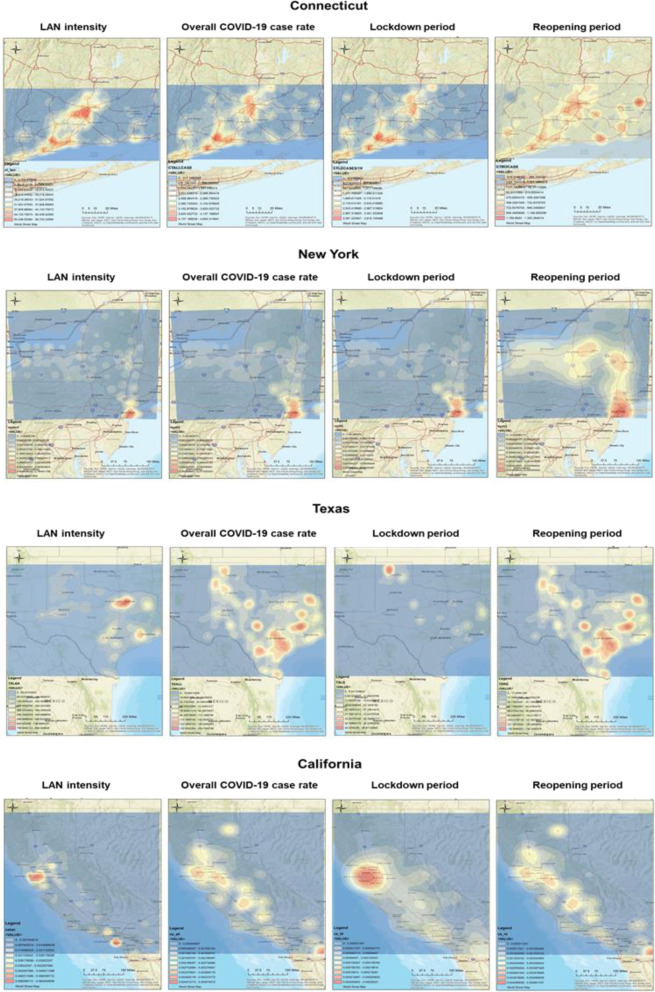
Co-distribution of light at night (LAN) and COVID-19 incidence in the US states of Connecticut, New York, Texas, and California. We used ArcGIS to generate the Kernel density maps of LAN intensity and COVID-19 case rate for these 4 states. There are significant positive correlations between LAN intensity and COVID-19 case rate in Connecticut and New York; no consistent correlation between LAN intensity and COVID-19 case rate in Texas and California. Data of LAN intensity were extracted from NASA Earth Observatory and COVID-19 case rates were obtained from local health departments

For Connecticut and New York, the hotspots of LAN intensity, COVID-19 case rates/1 k during lockdown, and COVID-19 case rates/1 k during the overall duration shared similar patterns that clustered around major cities in the state (Fig. 1), but were slightly different after reopening. Differently, in Texas and California, there were inconsistent patterns of hotspots for the four interested variables, but they share the trend that the hotspot-areas were similar for reopening and overall durations.

During the lockdown period, the hotspots of COVID-19 case rates shared similar patterns with LAN data in Connecticut and New York, but had different geo-patterns in California and Texas. During the reopening period, the locations of hotspots of COVID-19 case rates were very different from the LAN data map in New York, Texas, and California. The hotspot patterns of COVID-19 case rates during the overall period were similar to the LAN map in Connecticut and New York. On the contrary, the overall hotspots of COVID-19 case rates were similar to the reopening period in California and Texas.

**Fig. 2:**
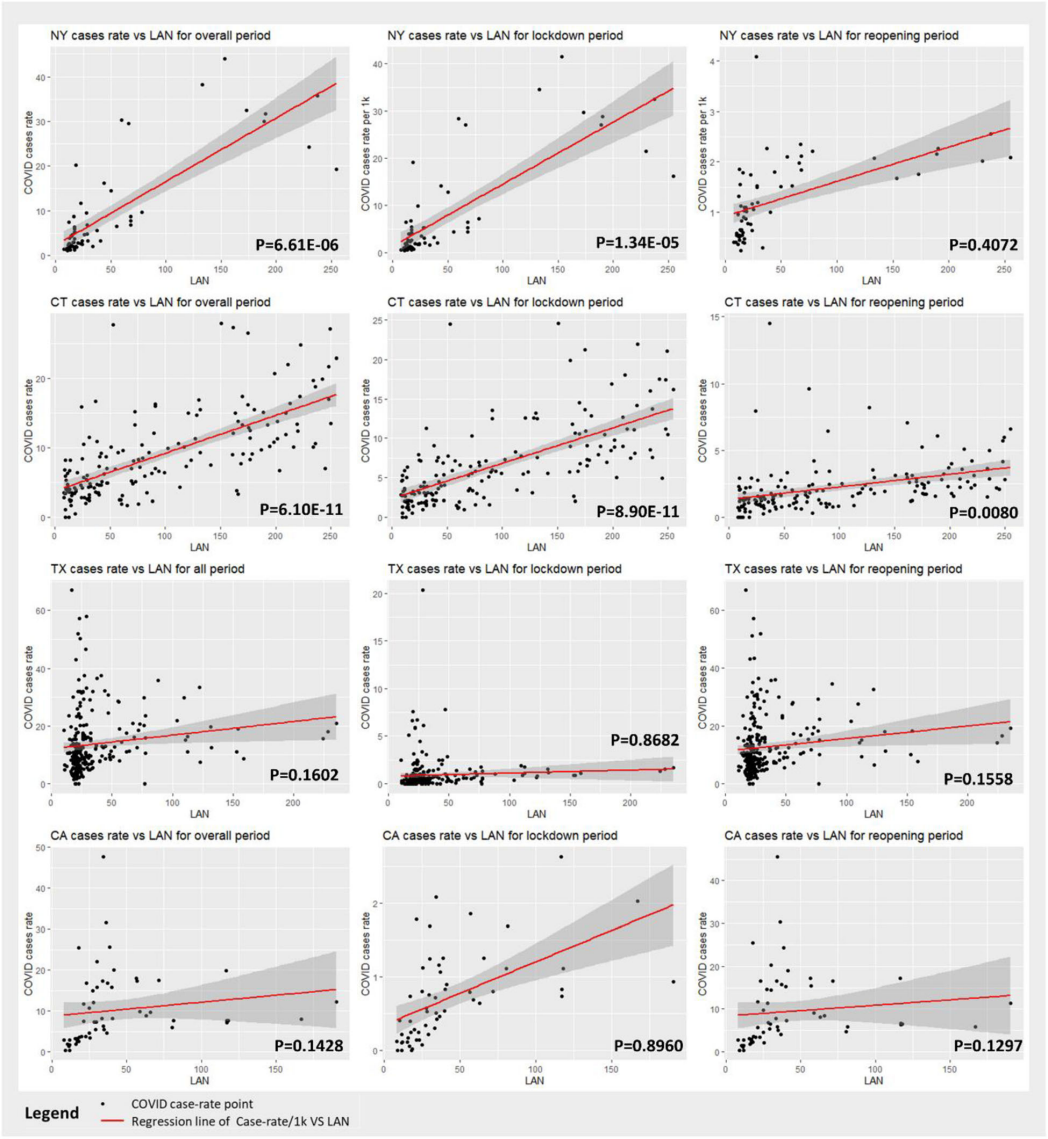
Scatter points of case-rate/1 k, LAN intensity and regression lines in the US states of Connecticut, New York, Texas, and California. *P* values indicate significance of association between COVID case-rate and LAN intensity from Regression models

An analysis of multivariable regression models also revealed similar patterns to the ones that geo-patterns showed. The cross comparison among four states showed that there were statistically significant correlations between LAN intensity and cases/1 k for the overall and lockdown durations in New York and Connecticut (*P* < 0.001). There was no statistically significant association between LAN intensity and cases/1 k, for the overall, lockdown, or reopening durations, in Texas and California (Table 1 and Fig. 2). The overall case rates were significantly associated with LAN in New York (*p* < 0.001) and Connecticut (*p* < 0.001), in which every 1 unit increase of LAN had a 15.6% increase in the overall case rate in New York, and a 3.7% increase in Connecticut. The results of the lockdown period were similar to those for the overall period in New York (*p* < 0.001) and Connecticut (*p* < 0.001). During the reopening period, there was a significant small positive association between case rates and LAN data in Connecticut (*p* < 0.001). Based on R-squared results, the state-specific regression models could explain more variations in New York (*R*^2^ = 0.80, 0.78 and 0.40) and Connecticut (*R*^2^ = 0.58, 0.57 and 0.21), compared to the data in California (*R*^2^ = 0.22, 0.37, 0.23) and Texas (*R*^2^ = 0.13, 0.02, 0.12), for the overall, lockdown and reopening durations.

**Table 1:** Summary table of Regression models of association between COVID case-rate and LAN intensity with covariant: nonwhite rate, percent below poverty, and population density for the overall, lockdown, and reopening periods for New York, Connecticut, California, and Texas

Variables	N	Overall cases rate			Lockdown cases rate			Reopening cases rate		
		Beta	***P***-value	R^**2**^	Beta	***P***-value	R^**2**^	Beta	***P***-value	R^**2**^
**New York**	62	0.156	6.61E-06	0.80	0.148	1.34E-05	0.78	0.003	0.4072	0.40
**Connecticut**	167	0.037	6.10E-11	0.58	0.030	8.90E-11	0.57	0.006	0.0080	0.21
**Texas**	254	0.043	0.1602	0.13	0.001	0.8682	0.02	0.041	0.1558	0.12
**California**	56	0.110	0.1428	0.22	0.001	0.8960	0.37	0.110	0.1297	0.23

N: county level data points for states of NY, CA, TX; town level data points for CT

Beta: beta-estimate for the regression model, representing the coefficient of models

R^2^: measured the goodness of fitting for regression models, representing percent of data that is able to be explained by the model

In addition, we also performed an increased regression model, as the sensitive analysis, with added covariants of education level, insurance coverage and the rate of disability in New York, California and Texas. We didn’t include Connecticut due to data unavailability. The increased models showed very similar results to the main models (Table 1) at a similar significant level. This additional analysis showed that the performance of the main model did not significantly improve when these factors were included.

## Discussion

Our findings demonstrate significant positive correlations between LAN intensity and COVID-19 case rates per 1000 in two of the four US states (Connecticut and New York) studied. These findings support the proposed hypothesis that high LAN exposures may disrupt circadian rhythms, which lead to a decrease in people’s immunity and consequently increase an individual’s vulnerability to infectious diseases such as COVID-19 [2].

The co-distribution of light at night (LAN) and COVID-19 incidence was more evident in New York and Connecticut. The regression models we built can explain more variations of COVID-19 data in New York and Connecticut than in Texas and California. This difference might be due to different lockdown/reopening policies in each state. For example, California had a shorter lockdown period, and there were no formal “Stay at Home” orders in Texas, compared to New York which has similar total COVID-19 cases and population [18–22]. In states with strict restriction policies, people spent much more time at home and made less movement during the pandemic, including traveling, recreation, grocery shopping, etc.; and much less people moved between different cities and states [27–29]. The less strict policies may introduce more social factors into the analysis that we cannot determine easily at this point. The more complex situation masks the correlation between LAN and COVID-19 infection. We also found some inconsistent results of the effect of factors of social determinants, which might be due to the different social and demographic structures in each state. Moreover, the different COVID-19 testing policies and availability in different states might introduce more variations into our analysis.

Data points for all four states combined are relatively less likely to have a very solid statistical analysis, but in order to discuss different policy impacts, we chose to analyze states because of the heterogeneity of each state. The data used in the study is at either town or county levels, which aggregate individual data into large spatial area levels and may introduce ecological fallacy. To reduce the effect of ecological bias, we performed GWR models for New York. This approach improved the analysis and generated a very high R-squared value from the regression models (Supplement Table 1).

The current analysis focused on the COVID-19 test rate, and information on the severity of infected patients were not included due to its unavailability. Confounding factors considered in the analysis were percent below poverty, non-white rate, and population density. The increased regression model with added covariant of education level, insurance coverage and the rate of disability showed very similar results to the main model, which shows that these factors might not play important roles in the association. More confounders, such as employment, underlying health conditions, and proximity to healthcare facilities should also be considered in future studies if they are available.

Observations from our study are consistent with findings from a recent study that shows melatonin usage is significantly associated with a 28% reduced likelihood of a positive laboratory test result for COVID-19 [30]. Melatonin production in the pineal gland is sensitive to light and it has shown that even exposures to low intensity light can suppress melatonin secretion [31]. LAN may reduce melatonin levels and consequently increase risk of COVID-19 infection. Based on our results and existing literature, decreasing unnecessary LAN exposure might reduce its adverse effect on human immunity. Increasing awareness of the health effects of LAN and changing daily behaviors can decrease the exposure of LAN, which might reduce the vulnerability of pandemic infection. Daily measures include using heavy curtains and sleep patches, and turning off unnecessary ambient lights, etc. Moreover, policy level systematic measures can largely decrease the LAN exposure in general ambient environments, such as turning off unnecessary high intensity lights and decoration lights.

Due to mental pressure, behavior, and daily routine changes during the pandemic, there are increasing concerns of sleep disturbances and circadian disruptions, especially for healthcare workers [5, 32, 33]. More circadian disruptions might lead to more adverse impacts on human immunity, such as causing people to become more vulnerable to infectious diseases and other hormone-related diseases. This provides more opportunities to analyze how circadian disruptions such as LAN correlate with hormone-related health outcomes and temporal immune dynamics [34].

## Conclusion

In summary, both LAN intensity and COVID-19 case rates are higher in major cities or metro-centers in all four states, due to the nature of cities of higher mobility, population density, etc. In the current study, we observed a significant positive correlation between LAN intensity and COVID-19 cases-rate/1000, which suggests that circadian disruption of ambient light may increase the COVID-19 infection rate possibly by affecting an individual’s immune functions. Furthermore, differences in demographic structure and lockdown policies in each state play an important role in COVID-19 infections.

## Supplementary Information

**Additional file 1 **: **Supplement Table 1.** Spatial Autocorrelation test (Global Moran’s I) of cases/1 k during lockdown, reopening and overall durations, and Geographically Weighted Regression of cases /1 k with variables: LAN2016, nonwhite rate, percent below poverty, and population density during lockdown, reopening and overall durations, for New York. **Note:** Moran’s Index: The tendency of geo-clustering or geo-dispersion. A positive Moran’s I show the tendency of geo-clustering; Z-score: the critical value for test under standard normal distribution; Bandwidth: distance band or neighbors used for each local regression equation; Residual squares: sum of squared residuals, smaller the measure, the closer the fit of GWR models to observed data; Sigma: square root of the normalized residual sum of squares represent standard deviation for residuals.

## Data Availability

We obtained COVID-19 cases and testing data from local health departments. Specifically, we obtained COVID-19 data for Connecticut from the Connecticut State Department of Public Health (https://data.ca.gov/dataset/covid-19-cases), for New York from the Open NY Program (https://data.ny.gov/browse?tags=covid-19), for Texas from Texas Health and Human Services (https://dshs.texas.gov/coronavirus/additionaldata.aspx), and for California from the California Open Data Portal (https://data.ca.gov/dataset/covid-19-cases). Data of COVID-19 cases were categorized in 3 groups: duration of overall period (March 20th, 2020 to August 24th, 2020), period of lockdown, and period of reopening in each state according to local state policy on their government websites. All of the above databases are open to the public and no permissions are required to access these data.

## Abbreviations

LAN: Light at night
SCN: Suprachiasmatic nucleus
GWR: Geographically Weighted Regression
KD: Kernel Density
DMSP: Defense Meteorological Satellite Program
