# Supplementary material for: Co-distribution of Light At Night (LAN) and COVID-19 incidence in the United States
Source: BMC Public Health. 2021 Aug 4;21:1509. doi: 10.1186/s12889-021-11500-6 (PMC8335974; doi:10.1186/s12889-021-11500-6)
Supplement: Supplementary file 1 — Additional file 1 : Supplement Table 1. Spatial Autocorrelation test (Global Moran’s I) of cases/1 k during lockdown, reopening and overall durations, and Geographically Weighted Regression of cases /1 k with variables: LAN2016, nonwhite rate, percent below poverty, and population density during lockdown, reopening and overall durations, for New York. Note: Moran’s Index: The tendency of geo-clustering or geo-dispersion. A positive Moran’s I show the tendency of geo-clustering; Z-score: the critical value for test under standard normal distribution; Bandwidth: distance band or neighbors used for each local regression equation; Residual squares: sum of squared residuals, smaller the measure, the closer the fit of GWR models to observed data; Sigma: square root of the normalized residual sum of squares represent standard deviation for residuals. [file 12889_2021_11500_MOESM1_ESM.docx]

**Supplement Table 1:** Spatial Autocorrelation test (Global Moran's I) of cases/1k during lockdown, reopening and overall durations, and Geographically Weighted Regression of cases /1k with variables: LAN2016, nonwhite rate, percent below poverty, and population density during lockdown, reopening and overall durations, for New York.

|  | **Lockdown cases rate** | **Reopening cases rate** | **Overall cases rate** |
| --- | --- | --- | --- |
| Spatial Autocorrelation (Global Moran's I) (Spatial Statistics) for data in New York State | | | |
| Moran's Index | 0.844 | 0.327 | 0.412 |
| Variance | 0.005 | 0.005 | 0.005 |
| Z-score | 11.81 | 4.71 | 6.11 |
| P-value | <0.0001 | <0.0001 | <0.0001 |
| Geographically Weighted Regression of COVID cases /1k in New York State | | | |
| Mean Coefficient (SD) | 0.070 (0.026) | 0.011 (0.0002) | 0.129 (0.024) |
| Bandwidth | 197257 | 609157 | 218270 |
| Residual squares | 562.9 | 14.91 | 997.09 |
| Sigma | 3.487 | 0.521 | 4.561 |
| R^2^ | 0.912 | 0.557 | 0.862 |
| R^2^ adjusted | 0.884 | 0.508 | 0.825 |

**Note:**

Moran’s Index: The tendency of geo-clustering or geo-dispersion. A positive Moran’s I show the tendency of geo-clustering;

Z-score: the critical value for test under standard normal distribution;

Bandwidth: distance band or neighbors used for each local regression equation;

Residual squares: sum of squared residuals, smaller the measure, the closer the fit of GWR models to observed data;

Sigma: square root of the normalized residual sum of squares represent standard deviation for residuals;
